# Spider Angioma Number and Location as Potential Prognostic Indicators in Chronic Liver Disease: A Case Report

**DOI:** 10.7759/cureus.34193

**Published:** 2023-01-25

**Authors:** Kristy Terp, Guillermo Izquierdo-Pretel

**Affiliations:** 1 Internal Medicine, Herbert Wertheim College of Medicine, Florida International University, Miami, USA

**Keywords:** prognosis, chronic liver disease, location, number, spider angioma

## Abstract

Spider angiomas (SAs) are a well-known physical exam feature found in patients with chronic liver disease. While SAs are thought to correspond with a higher risk of mortality in chronic liver disease (CLD) patients, only few studies have been done to assess the number and location of SAs as prognostic indicators. We present a case of a 64-year-old patient with decompensated CLD who was found to have three SAs on physical exam. The patient presented to the inpatient service at a community hospital in Miami, Florida. He had experienced previous esophageal varices banding, had a Model for End-Stage Liver Disease (MELD) score of 31, and needed large-volume paracentesis due to significant ascites. It was determined that he had a very poor prognosis and was in need of a liver transplant. We suggest that more research is necessary to determine if there is a prognostic importance to the number and location of SAs in patients with CLD, as earlier interventions could potentially lead to improvements in outcomes through this physical exam finding.

## Introduction

Spider angiomas (SAs) are vascular lesions found in the dermis layer of the skin just beneath the surface. They contain a central erythematous area with extensions that radiate outward like a spider’s web. SAs form when the sphincteric muscle that surrounds a cutaneous arteriole fails, resulting in dilation of the arteriole. They are well-known physical exam features associated with chronic liver disease (CLD). SAs have been associated with other conditions such as rheumatoid arthritis, thyrotoxicosis, pregnancy, and severe malnutrition; however, findings of multiple SAs have a 95% specificity for CLD [1,2].

Although not well studied, SAs are thought to correspond with a higher risk of mortality in patients with established CLD [1]. SAs have also been associated with a higher likelihood of esophageal varices and hepatic fibrosis [2]. Furthermore, the number of SAs in patients with CLD is thought to correlate with the severity of disease [1]. To our knowledge, there have only been a few studies that have looked at the association between SAs and the severity of CLD. While these studies have shown that SAs are associated with development of subcutaneous collateral vessels, and this vessel formation is associated with higher levels of vascular growth factors and higher Model for End-Stage Liver Disease (MELD) scores, there has been no consensus on the number and location of SAs and how these factors correlate with CLD severity and outcomes [3,4]. Here we present a case of CLD necessitating liver transplant in a patient found to have multiple SAs, and suggest the need for further research on the prognostic indicators of SA number and location in CLD patients.

## Case presentation

The patient was a 64-year-old male with CLD and cirrhosis resulting from nonalcoholic steatohepatitis who presented with decompensated cirrhosis of the liver. His medical history was further complicated by the following co-morbid conditions: hypertension, hyperlipidemia, diabetes, benign prostatic hyperplasia, and recurrent migraines. A month prior to presentation, he also had three large esophageal varices greater than 5 millimeters banded in the lower third of his esophagus. Upon admission, he reported symptoms of altered mental status, headache, nausea, mild chest pain, epigastric pain, diarrhea, and decreased appetite for 7-10 days. His MELD score was 31, indicative of an estimated 27%-32% 90-day mortality. The MELD score is calculated using serum bilirubin, serum creatinine, and international normalized ratio (INR), all of which were elevated in this patient [5].

**Figure 1:**
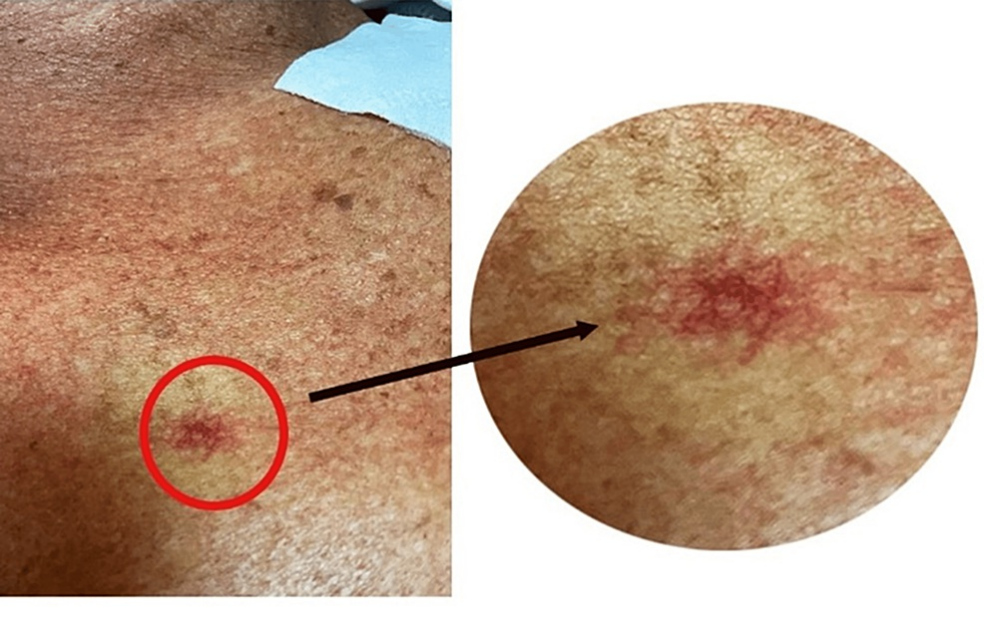
Spider angioma located on the anterior chest wall

**Figure 2:**
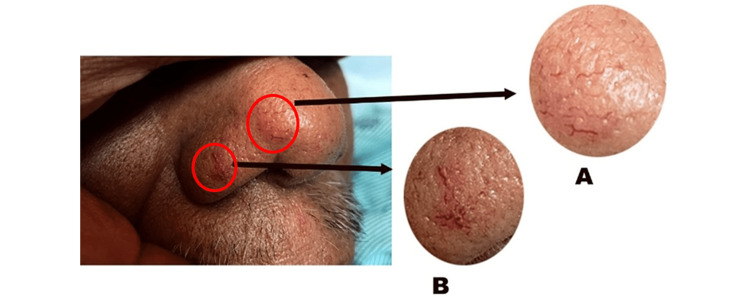
Two spider angiomas (A and B) located on patient’s nose

On physical examination, the patient was found to have significant ascites with shifting dullness. Three SAs were found: one on his anterior chest, and two on his nose (Figures 1, 2). No asterixis, palmar erythema, or jaundice was seen. The patient’s presentation of a large volume of ascites met the criteria for large-volume paracentesis while on the inpatient service, resulting in the removal of 8 liters of ascitic fluid. Culture of the ascitic fluid revealed extended-spectrum beta-lactamase-producing Escherichia coli that was treated with ertapenem.

Patient’s hepatic encephalopathy, with an initial ammonia level of 154 umol/L, was successfully treated with lactulose and rifaximin. Albumin, 50 grams per 200 milliliters, was given intravenously after large-volume paracentesis to prevent further circulatory dysfunction. Additional lab values throughout the patient’s hospital stay are shown in Table 1. After treatment on the inpatient service, the patient was discharged as stable and put on the list for liver transplant due to his CLD with recurrent complications.

**Table 1:** Serum markers in chronic liver disease from the time of admission to the time of discharge during the decompensated liver disease treatment BUN: blood urea nitrogen; AST: aspartate aminotransferase; ALT: alanine transaminase; INR: international normalized ratio; mmol/L: millimoles per liter; uL: microliter; g/dL: grams per deciliter; mEq/L: milliequivalents per liter; ng: nanogram; L: liter; umol/L: micromoles per liter Values in boldface are values that deviated outside the normal lab value range.

	Time of admission	Midpoint of the hospital stay	Time of discharge	Reference values
White blood cell count	2.55 x 10^3^/uL (low)	6.3 x 10^3^/uL	5.7 x 10^3^/uL	4.0–10.5 x 10^3^/uL
Red blood cell count	6.0 x 10^6^/uL (high)	2.8 x 10^6^/uL (low)	2.43 x 10^6^/uL (low)	4.2–5.6 x 10^6^/uL
Hemoglobin	9.1 g/dL (low)	9.7 g/dL (low)	8.6 g/dL (low)	13.3–16.3 g/dL
Hematocrit	24.7% (low)	27.4% (low)	24.1% (low)	39.0%–47.1%
Platelet count	96 x 10^3^ /uL (low)	74 x 10^3^/uL (low)	38 x 10^3^/uL (low)	140–400 x 10^3^/uL
Glucose	140 mg/dL (high)	169 mg/dL (high)	145 mg/dL (high)	74–106 mg/dL
Sodium	132 mmol/L (low)	138 mmol/L	130 mmol/L (low)	137–145 mmol/L
Potassium	3.2 mmol/L (low)	3.2 mmol/L (low)	3.2 mmol/L (low)	3.6–5.0 mmol/L
Chloride	103 mmol/L	100 mmol/L	95 mmol/L (low)	98–106 mmol/L
Carbon dioxide (CO_2_)	13 mEq/L (low)	23 mEq/L	31 mEq/L (high)	23–29 mEq/L
BUN level	62 mg/dL (high)	53 mg/dL (high)	50 mg/dL (high)	9–20 mg/dL
Creatinine level	5.3 mg/dL (high)	2.5 mg/dL (high)	1.56 mg/dL (high)	9.66–1.25 mg/dL
Calcium level	8.2 mg/dL (low)	9.8 mg/dL	8.0 mg/dL (low)	8.4–10.2 mg/dL
Total protein	7.2 g/dL	7.9 g/dL	5.4 g/dL (low)	6.0–8.2 g/dL
Albumin level	2.8 g/dL (low)	4.1 g/dL	2.5 g/dL (low)	3.9–5.0 g/dL
AST	37 units/L	34 units/L	30 units/L	15–46 units/L
ALT	17 units/L (low)	40 units/L	17 units/L (low)	21–72 units/L
INR	3.11 (high)	2.33 (high)	2.1 (high)	1.0
Alkaline phosphatase	152 units/L (high)	151 units/L (high)	73 units/L	44–147 units/L
Total bilirubin	2.8 mg/dL (high)	3.4 mg/dL (high)	5.2 mg/dL (high)	0.1–1.2 mg/dL
Ammonia	154 umol/L (high)	<9 umol/L	23 umol/L	15–45 umol/L

## Discussion

Although the exact pathophysiology of SAs has not been delineated, there have been multiple hypotheses. Substance P, alcohol, and estrogen are three substances thought to be contributing factors in the dilation of arterioles under the skin. These substances all exert dilatory effects on vasculature [1,2]. Another hypothesis is that angiogenesis leads to the formation of SAs through the production of vascular endothelial growth factor (VEGF) and basic fibroblast growth factor (bFGF) [6,7].

SAs are typically located on the face, neck, upper chest, and arms in adults. This distribution is thought to be related to the large vein that drains into the heart, the superior vena cava. In children, SAs tend to be more common on the upper extremities [1]. Similarly, in children with CLD, one study found that SAs were fewer and smaller when compared to findings in adults. Most children had less than five SAs (2.5% had more than five), very few were greater than 5 millimeters in size, and the distribution was mostly on the hands. In contrast, five or more SAs are thought to be more common in adults with CLD. Many normal children and pregnant women can also have findings of SAs, and they are more common in women compared to men [8,9]. Furthermore, while SAs are common to all types of CLD, they are especially common in patients with alcoholic cirrhosis [3,8,9].

When our patient presented with a physical exam finding of prominent ascites requiring large-volume paracentesis, closer attention to the physical exam directed our attention to three separate SAs: two on the patient’s nose and one on his anterior chest wall. The patient suffers from CLD and at our inpatient service, presented with decompensation. His history of recurrent ascites requiring paracentesis, hepatic encephalopathy, three large, previously banded esophageal varices, and a MELD score of 31 helped to determine that his overall prognosis was very poor, necessitating eventual liver transplant. Given what is already known about the pathophysiology and distribution of SAs, our patient’s case prompted us to further investigate previous studies that have been done that look at prognostic indicators in CLD patients, including the number and location of SAs.

Li et al. performed a study looking at the impact of SAs and subcutaneous collateral vessel formation of the chest/abdominal wall on the outcomes of liver cirrhosis [3]. In their study consisting of 198 patients with a median follow-up of 350 days, they found that the prevalence of SAs and subcutaneous collateral vessels of the chest/abdominal wall in cirrhotic liver patients was 47% and 29.8% respectively. They also found that SAs and subcutaneous collateral vessels of the chest/abdominal were significantly associated with higher MELD scores (10.77 ± 6.76 vs. 7.68 ± 5.42, p = 0.003), indicating a higher likelihood of mortality. Of the 93 patients with SAs, 49.46% (46/93), 21.51% (20/93), and 29.03% (27/93) had one to two, three to four, and five or more spider nevi, respectively. The location of the spider nevus was the chest wall alone in nearly all patients (97.85%, 91/93). Interestingly, they concluded that those with SAs did not have a significantly different cumulative survival compared to those with cirrhotic liver disease without SAs (p = 0.951). However, those with subcutaneous collateral vessels of the chest/abdominal wall had significantly worse cumulative survival than those without (p = 0.018). It is important to note that while there was not a significantly different cumulative survival in those with SAs, this was a relatively small study population with nearly half of the patients having only one to two SAs and moderately high MELD scores. This further raises the question of whether a larger study of patients with more severe MELD scores and a higher number of SAs would yield more significant results in terms of CLD prognosis and outcomes.

Li et al. did a study investigating whether VEGF and bFGF were associated with the formation of SAs in patients with cirrhotic liver disease [4]. In their study of 86 cirrhotic liver patients and 53 controls, the number and size of SAs were recorded and plasma levels of VEGF and bFGF were measured. They found that plasma levels of VEGF and bFGF were increased in cirrhotic liver disease patients (122 ± 13 vs. 71 ± 11 pg/mL, p = 0.003, for VEGF; 5.1 ± 0.5 vs. 3.4 ± 0.5 pg/mL, p = 0.022, for bFGF). Not only were these levels higher overall in cirrhotic liver patients compared with controls, but VEGF and bFGF levels were also higher in patients with SAs compared to those without (185 ± 28 vs. 90 ± 10 pg/mL, p = 0.003, for VEGF; 6.8 ± 1.0 vs. 4.1 ± 0.5 pg/mL, p = 0.017, for bFGF). Furthermore, plasma VEGF levels were negatively correlated with liver function reserve in liver cirrhosis patients. Early age was an additional factor that was found to be predictive of SA formation in patients with cirrhosis. Similar to the study by Li et al., this study with a relatively small study population did not look specifically at the number and location of SAs and their association with prognosis in CLD patients. However, the finding of increased levels of VEGF and bFGF in CLD patients with SAs, and the associated decreased liver function reserve suggests a worse prognosis.

While these previous studies have established an association between the presence of SAs and CLD severity, there is no clear evidence that looks specifically at the association of number and location of SAs as prognostic predictors in patients with CLD. Furthermore, our study has the limitation of only looking at one patient with total three SAs located on the nose and chest wall, which is less than the commonality of five or more SAs found in adults [8]. While our patient did have severe CLD with an overall poor prognosis, these case report findings provide poor external validity in regard to SA number and location associated with CLD severity, as our patient also had multiple co-morbidities that could complicate his CLD, and not all CLD patients have such co-morbidities. Nonetheless, we suggest that the location and number of SAs could potentially be used as indicators of disease severity and prognosis in CLD patients given previous research findings and findings in our patient, particularly, the high MELD score and the need for liver transplant in the setting of multiple visible SAs. Similarly, as H Li et al. and CP Li et al. suggested, subcutaneous collateral vessels of the chest/abdominal wall and plasma levels of VEGF and bFGF are additional markers of prognosis in CLD patients that have the potential to indicate disease severity and need for earlier intervention [3,4]. More research with larger-scale studies needs to be done that looks at the association between these factors and overall prognosis in CLD patients, which could potentially lead to improved outcomes through earlier interventions.

## Conclusions

Here, we have presented a case of a patient with three SAs, one on the anterior chest and two on the nose. This patient who presented with decompensated liver failure with a high MELD score of 31 was ultimately determined to have a very poor prognosis necessitating liver transplant, raising the question of whether or not his visible SAs had any correlation with his CLD severity. Given that this case report is limited by the study of one patient with multiple co-morbidities, we suggest more research to be done to determine if there is a prognostic importance to the number and location of SAs in patients with CLD. Further studies could help delineate if the total number of SAs and their location are associated with CLD severity. This could be simple, yet powerful, physical examination finding that prompts the investigation of CLD status before it progresses to end-stage liver disease. Plasma levels of VEGF and bFGF also have the potential to be used as prognostic markers in patients with CLD. These quantitative and qualitative markers could potentially lead to earlier interventions through the determination of the degree of severity of CLD.
